# An Unusual Case of H3K27-Altered Diffuse Midline Glioma Presenting as a Third Ventricular Mass

**DOI:** 10.7759/cureus.82035

**Published:** 2025-04-10

**Authors:** Hayley Mitchel, Trevor Huff, Kirsten Pickard, Raymond Carmody, Hasan Ozgur, Alexis Elliott, Samuel Rogers

**Affiliations:** 1 Radiology, Midwestern University Arizona College of Osteopathic Medicine, Glendale, USA; 2 Radiology, University of Arizona College of Medicine - Tucson, Tucson, USA; 3 Pathology, University of Arizona College of Medicine - Tucson, Tucson, USA; 4 Neuroradiology, University of Arizona College of Medicine - Tucson, Tucson, USA; 5 Diagnostic Radiology, University of Arizona College of Medicine - Tucson, Tucson, USA; 6 Neuroradiology, Radiology Ltd., Tucson, USA

**Keywords:** an unusual case, cns who grade 4 tumor, diffuse midline glioma, neuro ct, neuro mri, neuroradiology, unusual location

## Abstract

Diffuse midline gliomas (DMGs) are rare central nervous system tumors, typically arising from astrocytes. The H3K27-altered DMG subtype is defined by a specific histone protein mutation that disrupts normal cellular regulation and promotes unchecked proliferation. Due to their aggressive nature, the World Health Organization (WHO) universally classifies these as grade IV, regardless of histological appearance. While DMGs are most commonly described in pediatric populations and overwhelmingly originate in the pons, their occurrence and clinical progression in adults remain underexplored. This report highlights a rare presentation of an H3K27-altered DMG, presenting as a third ventricular mass in a 30-year-old man, emphasizing its atypical location and the challenges it presents for diagnosis and management.

## Introduction

Diffuse midline gliomas (DMGs) are a rare and aggressive subtype of glial tumors that primarily affect the midline structures within the central nervous system. Globally, an estimated 6.8 per 100,000 people are diagnosed with DMGs each year, with glioblastomas accounting for 75% of these tumors [1]. While DMGs have been widely studied, there is a paucity of data specific to the H3K27-altered subtype. First recognized as a distinct pathological entity in the 2016 World Health Organization (WHO) classification of central nervous system tumors, these tumors were initially termed DMG H3K27M and later updated to H3K27-altered DMG [2-4]. Characterized by their midline location, diffuse infiltrative growth pattern, and the presence of an H3 K27M-specific mutation, H3K27-altered DMGs are universally classified as WHO grade IV tumors, underscoring their poor prognosis.

H3K27-altered DMGs are predominantly observed in pediatric populations, with significantly fewer cases documented in adults [5]. These tumors typically arise from astrocytes and are most commonly located in the pons and thalamus. However, they may also originate in other midline regions such as the medulla and spinal cord [6-8]. While histological, genetic, and radiological evaluations can help guide prognosis, these tumors are associated with an average life expectancy of less than one year [9,10]. While the H3K27-altered mutation is detected in approximately 80% of pediatric DMGs, its prevalence and progression in adults remain less well-defined [11].

The presenting symptoms of DMGs vary depending on the tumor's location. Supratentorial lesions frequently result in hydrocephalus-related symptoms, including headaches, hemiplegia, nausea, and vomiting. Infratentorial tumors, in contrast, more commonly manifest with cranial nerve palsies, sensory or motor dysfunction, and cerebellar symptoms such as ataxia and nystagmus [4,8,12]. As these tumors infiltrate surrounding brain tissue, they may disseminate to other regions of the central nervous system via cerebrospinal fluid (CSF), further complicating treatment. Due to their complex anatomical location, surgical resection is typically unfeasible. Standard management usually includes glucocorticoids to reduce peritumoral edema alongside chemoradiation to slow tumor progression [3,8,13].

Despite the recognition of H3K27-altered DMGs in both pediatric and adult populations, critical gaps in understanding its incidence, clinical presentation, and outcomes in adults persist. This report contributes to this knowledge by describing a rare case of an adult with H3K27-altered DMG, highlighting the diagnostic and therapeutic challenges posed by this condition.

## Case presentation

**Figure 1:**
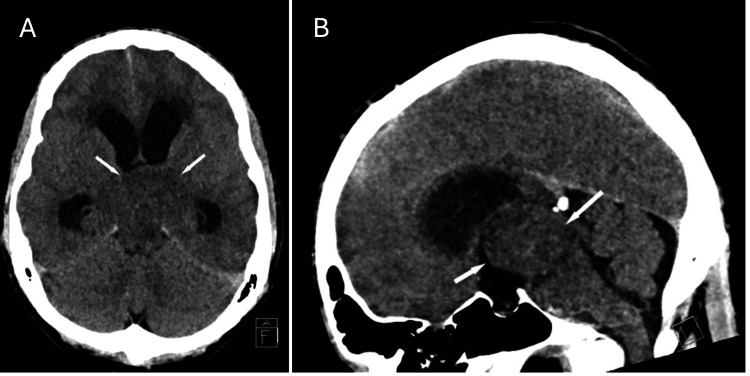
Non-contrast CT at admission (A) Axial and (B) sagittal images. Arrows indicate a mass isodense to white matter filling the third ventricle, with associated obstructive hydrocephalus. CT: computed tomography

**Figure 2:**
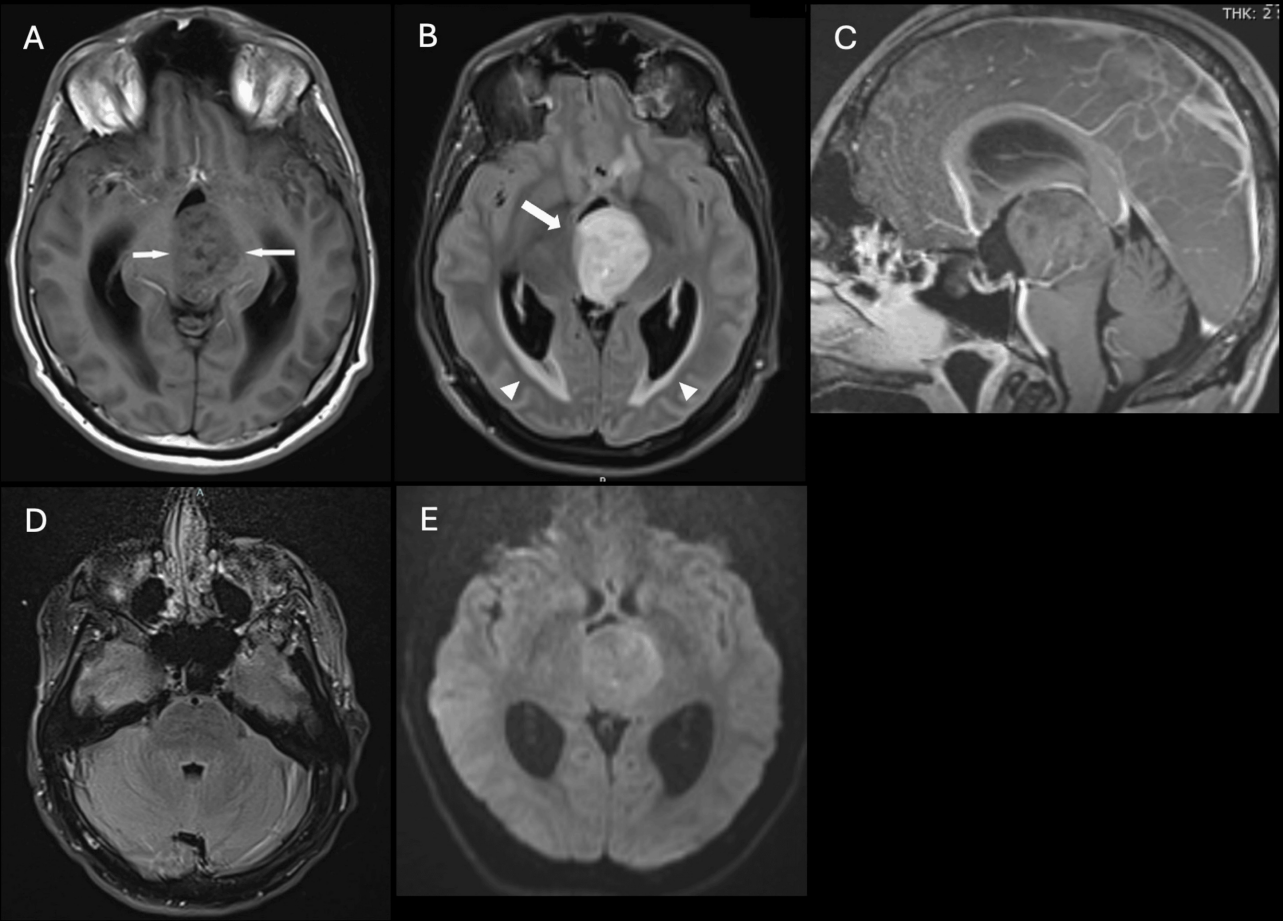
Tumor baseline MRI MRI of the midline mass measuring 3.3 × 3.7 × 3.1 cm at index hospitalization. (A) Axial non-contrast T1W image shows an intermediate signal, heterogeneous third ventricular mass (arrows). (B) Axial FLAIR image shows homogeneous T2 hyperintensity (arrow). Note transependymal CSF migration surrounding the occipital horns related to obstructive hydrocephalus (arrowheads). (C) Sagittal gadolinium-enhanced image shows minimal enhancement of the mass, occupying the third ventricle and invading the thalamus and mesencephalon. (D) Axial FLAIR image of the pons shows no tumor involvement. (E) Diffusion-weighted images demonstrated no significant restricted diffusion. MRI: magnetic resonance imaging, FLAIR: fluid-attenuated inversion recovery, CSF: cerebrospinal fluid

A 30-year-old man presented with a four-day history of headache, nausea, vomiting, and presyncope. Upon arrival at the emergency department, he experienced an acute change in mental status, characterized by horizontal beating nystagmus, gaze deviation, and upper extremity posturing. Computed tomography (CT) revealed a large isodense mass at the level of the third ventricle, causing severe obstructive hydrocephalus with bilateral lateral ventricle enlargement and transependymal CSF flow (Figure 1). A right-sided external ventricular drain (EVD) was placed to alleviate intracranial pressure. Subsequent magnetic resonance imaging (MRI) showed a mass involving the posterior third ventricle, thalamus, and mesencephalon (Figure 2). It was T1 isointense and T2 hyperintense, with mild heterogeneous enhancement and no significant diffusion restriction. The patient underwent an endoscopic third ventriculostomy and biopsy. The EVD was removed a few days later, and he was discharged home.

**Figure 3:**
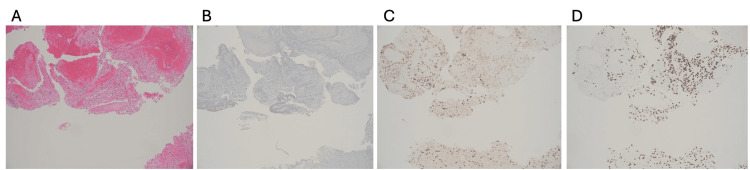
Third ventricle mass biopsy (A) Hypercellular neoplasm with nuclear atypia (H&E, 10×). (B) Negative for IDH-1 mutation (10×). (C) Decreased me3 staining (10×) indicates widespread loss of H3K27 methylation on H3 histones. (D) Positive H3K27M immunohistochemistry (10×) confirms the presence of the mutated H3K27 protein. Final diagnosis: H3K27-altered DMG, WHO grade IV. H&E: hematoxylin and eosin, DMG: diffuse midline glioma, WHO: World Health Organization

Histological examination revealed a hypercellular astrocytic neoplasm with nuclear atypia but without necrosis or vascular proliferation (Figure 3). Molecular analysis confirmed the presence of the H3F3A.p.K28M mutation, consistent with a diagnosis of H3K27-altered DMG, WHO grade IV. Additional findings included mutations in p53 and the C250T TERT promoter. Analysis for further mutations was negative (Table 1).

**Table 1:** Immunohistochemical and molecular profiling of third ventricle mass biopsy

Biomarker	Result
H3F3A	Pathogenic variant exon 2
p53	No mutation detected
IDH1/IDH2	No mutation detected
TERT promoter	Pathogenic variant C250T
BRAF	No mutation detected
MSI	Stable
NTRK1/2/3	Fusion not detected
RET	Fusion not detected
Tumor mutational burden	Low
ATRX	No mutation detected
CDKN2A/CDKN2B	No mutation detected
MGMT	Unmethylated

The patient underwent 42 days of temozolomide therapy (75 mg/m^2^ daily) with concurrent radiation therapy (54 Gy in 30 fractions of 1.8 Gy/fraction), followed by six additional 28-day cycles of maintenance temozolomide (200 mg/m^2^). He was also treated with tumor treating electrical field therapy (TTF). Throughout treatment, the patient remained symptomatically stable, and follow-up imaging demonstrated no significant progression of the mass.

**Figure 4:**
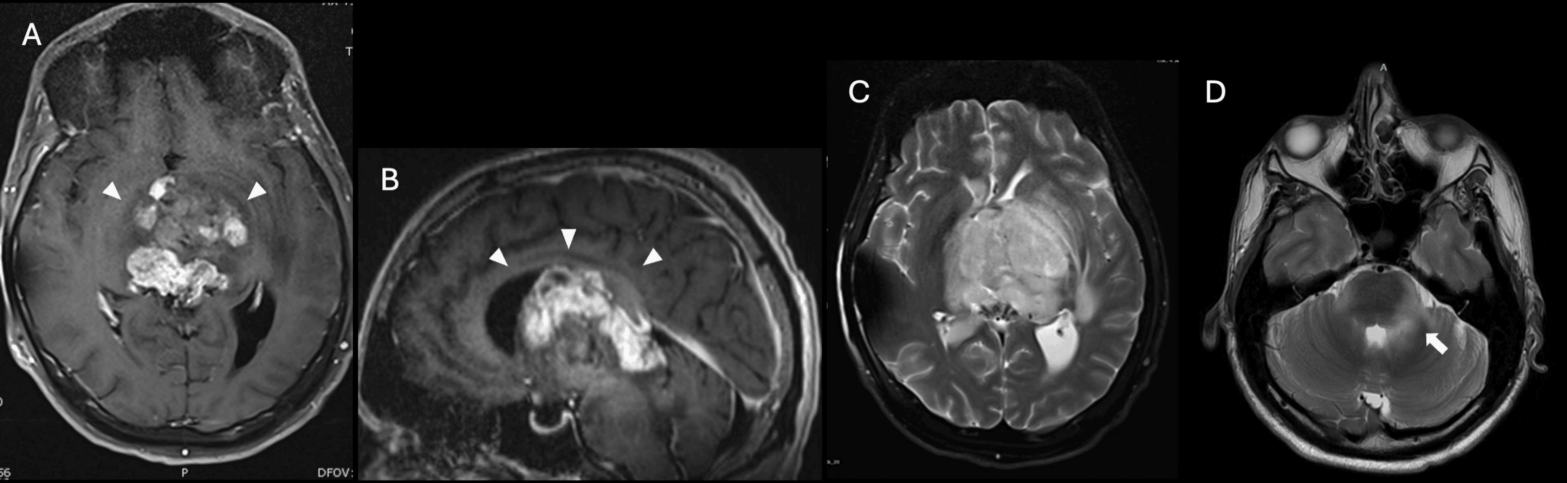
Tumor progression on MRI Follow-up MRI performed 11 months after diagnosis with interval increase in mass size to 4.6 × 5.1 × 5.2 cm. (A) Axial and (B) sagittal T1W enhanced images show an increase in tumor size throughout the third ventricle and increased regions of enhancement, with extension into the basal ganglia, tectum, and corpus callosum (arrowheads). (C and D) Axial T2W images show heterogeneous T2 signal with severe local mass effect and invasion into the bilateral thalami, left basal ganglia, and new extension into the pons and left middle cerebellar peduncle (arrow). MRI: magnetic resonance imaging

Soon after completing his last cycle of chemotherapy and 11 months after diagnosis, the patient had a seizure and was hospitalized. A ventriculoperitoneal shunt was placed, and a repeat MRI showed progression of the disease with involvement of the basilar cisterns, pons, and midbrain (Figure 4). He was stabilized and discharged home at his baseline mentation on dexamethasone, bevacizumab, and levetiracetam.

Unfortunately, he was readmitted a week later due to worsening encephalopathy. Despite supportive care, he became progressively somnolent and died two weeks later, 12 months after his initial diagnosis.

## Discussion

H3K27-altered DMGs are a rare and highly aggressive subtype of glial tumor associated with poor outcomes. Their defining feature is a specific mutation in which lysine at codon 27 is substituted with methionine, leading to hypomethylation of the histone coding region and inhibition of polycomb repressive complexes [3]. This mutation predominantly occurs on the H3F3A gene (70% of cases) and less commonly on HIST1H3B and HIST1H3C [11,14]. The mutation confers high-grade behavior to the tumor, even in the absence of high-grade histological features such as high mitotic activity or necrosis. Life expectancy for patients with H3K27-altered DMGs is significantly shorter than for those with DMGs lacking the mutation (0.73 versus 4.6 years) due to the extensive gene expression dysregulation driven by this mutation [15,16].

While H3K27-altered mutations have been identified in adult DMGs, they are far less common than in pediatric cases. It is reported to occur in 15%-60% of adult DMGs versus 80% of pediatric DMGs [11,17,18]. Furthermore, the prevalence and clinical implications of this mutation in adults remain poorly understood. Current evidence suggests that adult-onset DMG H3K27-altered tumors may be associated with a slightly but significantly more favorable prognosis compared to pediatric cases [5,12]. However, the pathological factors underlying this difference require further investigation.

Distinctive imaging features for H3K27-altered DMGs have not been identified, as findings are highly variable [12]. On MRI, these lesions appear T1 hypointense and T2 hyperintense. They typically show facilitated diffusion, although regions of diffusion restriction may correspond to higher-grade areas [12,19]. Enhancement patterns vary widely, ranging from non-enhancing to patchy or ring-like enhancement. Edema, necrosis, and hemorrhage are inconsistently observed [4,11,12,19]. On CT, tumors generally appear hypo- or isodense with minimal or no enhancement. Tumor progression is marked by increasing diffusivity and infiltration.

Of note, this case is an unusual example of an H3K27-altered DMG appearing to originate from the third ventricle. Imaging findings of tumors more commonly associated with the third ventricle (such as colloid cysts, choroid plexus papillomas, and ependymomas) are markedly distinct from those seen in DMGs, which can aid in narrowing the differential diagnosis. However, the imaging findings of H3K27-altered DMG closely resemble those of other high-grade gliomas, and no distinctive imaging features have been identified for DMGs. As a result, a biopsy is often necessary for a definitive diagnosis, but this is challenging and sometimes dangerous due to the tumors' diffuse midline distribution and potential for hemorrhage. This increases the risk of misdiagnosis and complicates prognostic accuracy.

By definition, DMGs are located within midline structures, with the pons being the most common site for H3K27-altered tumors, followed by the thalamus and spinal cord [7,8,14]. In adults, the thalamus is the most frequently affected location [12]. Lesions predominantly involving the third ventricle, as in this case, are exceedingly rare and likely originate from the thalamus [6-8,19]. Notably, however, the majority of DMGs presenting in the third ventricle are positive for the H3K27-altered mutation [6]. DMGs may initially present with a diffuse appearance or appear more mass-like, progressively adopting a more diffuse pattern as the disease advances. They lack well-defined borders, infiltrate surrounding structures, and frequently cross midline pathways via white matter tracts. Their midline location and infiltrative nature pose significant challenges to treatment, as surgical resection is typically not feasible.

The genetic composition of adult H3K27-altered DMGs has not been explored in depth. Studies have investigated mutations in a range of proto-oncogenes and tumor suppressor genes, including IDH1/2, CDKN2A/B, ATRX, TP53, TERT, NF1, MGMT, and PPM1D [4,8,9]. Mutations in these genes are generally rare in adult cases, with the exception of ATRX, a tumor suppressor gene altered in up to 38% of tumors and linked to poorer survival rates. TERT mutations, as observed in this case, drive tumorigenesis by increasing telomerase activity. Although not widely reported in H3K27-altered DMGs, these mutations are generally associated with decreased survival in other gliomas, although the specific mechanisms that drive survival and treatment response in relation to these mutations are not well understood [20]. MGMT encodes an enzyme that repairs DNA damage caused by alkylating agents. MGMT methylation suppresses the transcription of this enzyme, which enhances tumor sensitivity to alkylating chemotherapeutic agents [1]. Further identifying specific biomarkers within H3K27-altered DMGs could help guide targeted therapies and promote the development of novel treatment strategies.

Currently, no curative therapies exist for H3K27-altered DMG, and treatment primarily focuses on extending survival and managing symptoms [3,8,13]. Traditionally, treatment involves a combination of radiotherapy and chemotherapy and surgical debulking if the tumor location permits; however, no specific treatment guidelines currently exist.

Experimental treatments for DMGs are currently under investigation, including new techniques for enhanced drug delivery, genomic medicine, and immunotherapies. Several mechanisms are being explored for targeted drug delivery. Magnetic resonance-guided focused ultrasound (MRgFUS) enhances the permeability of the blood-brain barrier, allowing for more precise delivery of chemotherapeutics, while convection-enhanced delivery (CED) uses positive pressure to drive drug solutions directly to the tumor site, bypassing the blood-brain barrier [21].

Various genetic mutations are being studied as potential targets for new treatments. Activin receptor-like kinase 2 (ALK2) inhibitors may offer a therapeutic option for tumors with the ACVR1 receptor tyrosine kinase mutation. Recently, the Food and Drug Administration (FDA) approved a combination therapy of dabrafenib and trametinib for pediatric patients with low-grade gliomas, which may also benefit patients with high-grade gliomas [21]. Additionally, drugs targeting the H3 mutation in H3K27-altered DMGs, such as GSKJ4, have demonstrated promising results in preclinical trials, particularly when used in combination with histone deacetylase inhibitors [21]. Antibody-drug conjugates targeting the epidermal growth factor receptor (EGFR) have been evaluated, but their clinical benefit has been limited, with significant toxicity to patients [22].

Immunotherapies, including vaccines and antibodies, are showing potential in clinical trials. Mueller et al. (2020) [23] reported a survival benefit in patients receiving an H3.3K27-altered-targeted vaccine, and Gállego Pérez-Larraya et al. (2022) [24] observed encouraging results using a modified oncolytic adenovirus vaccine for patients with the H3K27-altered mutation. Tumor-infiltrating lymphocyte (TIL) therapy has also been explored as a treatment for H3K27-altered DMGs. While promising, TIL therapy requires sufficient tissue samples via biopsy, which can be challenging due to the midline location of DMGs, making adequate sampling difficult [21].

The use of TTFs, as demonstrated in this case, can also serve as an adjunct therapy to inhibit tumor cell division. While this approach has been shown to extend survival, its effectiveness is inherently constrained by the limited ability of electrical fields to penetrate the skull and reach the target tissue [21]. Despite ongoing research into novel therapies, H3K27-altered DMG remains an aggressive tumor with few effective treatment options.

## Conclusions

The H3K27-altered mutation characterizes a distinct and aggressive subset of DMGs associated with limited treatment options and poor survival outcomes. Identification of this mutation, along with related genetic biomarkers and imaging features, is essential for advancing therapeutic strategies. This case of a third ventricle H3K27-altered DMG in a 30-year-old man represents a rare presentation, both in terms of its age of onset and atypical location. It illustrates the unpredictable nature of these tumors, remaining symptomatically stable for almost a year before experiencing a sudden and rapid deterioration. It also highlights the challenges in diagnosing and classifying these tumors. Although initially identified as a mass originating from the third ventricle, follow-up imaging and histological analysis suggested that it was more likely of thalamic origin. However, follow-up testing, as illustrated in this case, is often not feasible due to the challenges of biopsying midline locations and the typically rapid progression of symptoms. This case underscores the challenges posed by the tumor's rarity, midline origin, and infiltrative nature, emphasizing the critical need for ongoing research and innovative treatment strategies.
